# Development and Characterization of Organosilicon-Based Asphalt Wearing Course with Enhanced Erosion and Skid Resistance for Low-Carbon Pavement Maintenance

**DOI:** 10.3390/ma19142941

**Published:** 2026-07-08

**Authors:** Yu Song, Jianlin Feng, Wei Liu, Haiqin Xu, Shaopeng Wu, Lei Zhang

**Affiliations:** 1 State Key Laboratory of Silicate Materials for Architectures, Wuhan University of Technology, Wuhan 430070, Chinafengjianlin@whut.edu.cn (J.F.); liuw321@whut.edu.cn (W.L.); lei.zhang@chd.edu.cn (L.Z.); 2 Hubei Industrial Construction Group Co., Ltd., Wuhan 430076, China; 3 School of Materials Science and Engineering, Chang’an University, Xi’an 710064, China

**Keywords:** asphalt wearing course, organosilicon, durability, skid resistance, carbon emission

## Abstract

Asphalt pavement wearing courses are directly exposed to hydrodynamic scouring, fuel erosion, freeze–thaw action, and traffic abrasion, leading to accelerated surface deterioration, skid-resistance loss, frequent maintenance, and increased life-cycle carbon emissions. To address these challenges, this study developed an organosilicon-based erosion- and skid-resistant asphalt wearing course (OES-AWC) through a stepwise material design strategy. An organosilicon-treated asphalt concrete matrix was first prepared to improve resistance to moisture damage, fuel erosion, and ice adhesion, and its curing behavior and optimal dosage were determined. A skid-resistant surface layer was then designed by optimizing the anti-skid aggregate type, organosilicon-to-aggregate ratio, and surface texture. Finally, waterborne epoxy resin was introduced to enhance aggregate anchorage, and the integrated OES-AWC was evaluated in terms of abrasion durability, rutting resistance, long-term skid resistance, and life-cycle impacts. The results show that organosilicon treatment forms a hydrophobic siloxane network, which improves the moisture damage, fuel erosion, and anti-icing resistance of asphalt concrete by 22.0–41.1%. Emery aggregates and the optimized surface structure enhance friction stability, while waterborne epoxy resin significantly suppresses aggregate stripping under repeated wheel loading. Compared with conventional asphalt wearing courses, the optimized OES-AWC increased wear durability by 148.1% while maintaining stable skid resistance under prolonged abrasion. Life-cycle assessment further demonstrates that OES-AWC can reduce carbon emissions by 47.2% and overall costs by 25.0%, with a probability exceeding 90% according to the uncertainty analysis. These findings indicate that OES-AWC provides a durable, low-carbon, and cost-effective maintenance strategy for asphalt pavements exposed to complex service environments.

## 1. Introduction

Asphalt pavements are widely utilized in highways, port infrastructures, and heavy-duty industrial corridors due to their ease of construction, driving comfort, and noise reduction capabilities [1,2,3]. However, as the outermost layer directly subjected to vehicular loads and environmental stressors, the asphalt wearing course is continuously exposed to the synergistic effects of hydrodynamic scouring [4], fuel erosion [5], freeze–thaw cycles [6], and heavy traffic loading [7]. Consequently, it is highly susceptible to distresses such as moisture-induced damage, oil-erosion softening, aggregate stripping, and the degradation of anti-skid performance. Under high-temperature and high-humidity conditions, or within specialized service environments (e.g., port loading zones and logistics park transit hubs), these deterioration mechanisms are significantly accelerated. This accelerated degradation not only severely shortens the service life of the pavement but also poses a direct threat to traffic safety [8].

Moisture-induced damage represents one of the most critical durability challenges for asphalt mixtures [9]. Hydrodynamic pressure accelerates the penetration of moisture along the asphalt-aggregate interface, weakening interfacial adhesion and inducing stripping failures [10]. In addition to moisture-induced damage, asphalt pavements in specialized sections—such as port roadways and entrances to large-scale construction sites—are highly vulnerable to fuel erosion [11]. At the micro-level, oil erosion weakens the adhesion at the asphalt-aggregate interface and reduces the interfacial fracture energy, thereby increasing the susceptibility of the mixture to stripping and raveling [12]. Such aggressive service environment, compounded by heavy traffic loads, severely shortens the lifespan of the asphalt pavement. It triggers a vicious cycle of frequent maintenance and repairs that still fail to meet load-bearing demands, resulting in substantial economic costs and elevated carbon emissions [13,14].

Beyond moisture-induced damage and the vulnerabilities of specialized roadway sections, ice formation on asphalt pavements in low-temperature environments poses a critical threat to both traffic safety and structural durability [15,16]. Surface icing fundamentally alters the tire-pavement contact mechanics, transitioning the interface from a high-friction rough solid interaction to a low-shear-strength rubber–ice contact [17,18]. This transition masks the macro- and micro-texture of the pavement surface, resulting in a precipitous drop in the friction coefficient and severely compromising vehicular safety. Concurrently, the mechanical interlocking generated by ice crystal growth and the expansive stresses associated with freeze–thaw cycles significantly diminish the adhesion strength at the asphalt-aggregate interface. Under the shearing action of vehicular traffic, these stresses induce surface stripping and facilitate the propagation of microcracks, thereby accelerating the degradation of structural durability. To elucidate this phenomenon, Wang et al. [17] utilized acoustic emission (AE) characteristic parameters and digital image correlation (DIC) strain contour maps to investigate the failure evolution of asphalt mixtures subjected to varying freeze–thaw cycles. Their findings revealed that freeze–thaw action significantly reduced the AE energy and b-value of the asphalt mixtures by approximately 50% and 70%, respectively, resulting in the extensive generation of microcracks within the specimens. The subsequent infiltration of moisture and other erosive media into these microcracks further exacerbates the deterioration, ultimately culminating in severe asphalt stripping at the asphalt-aggregate interface.

In summary, the fundamental cause of interfacial weakening and structural degradation—whether induced by moisture damage, oil erosion, or low-temperature icing—stems from the direct contact and continuous interaction between external erosive media and the surface or internal interfaces of the asphalt concrete [19,20]. Consequently, constructing a continuous, stable protective barrier with low surface energy on the asphalt pavement surface to isolate internal interfaces from erosive media is a promising strategy for the synergistic mitigation of multi-source environmental degradation. However, surface treatments primarily function as an external shield; they are intrinsically limited in their ability to penetrate and encapsulate the complex network of internal interfaces deep within the asphalt mixture. Since interconnected pores and micro-cracks inevitably exist within the pavement structure, erosive agents such as moisture and fuel can infiltrate beyond the surface layer, directly attacking the internal asphalt-aggregate interfaces that remain unprotected. This inherent limitation underscores the critical need to develop modification methods capable of internally wrapping these internal interfaces. Organosilicon materials, characterized by their unique molecular structures and interfacial chemical properties, offer a novel technical approach to achieving this objective [21]. In the presence of moisture, alkoxy-containing organosilicon monomers undergo hydrolysis and polycondensation reactions to form a stable, -Si-O-Si- cross-linked network. Furthermore, these monomers can chemically bond with mineral surfaces via silanol groups, thereby constructing a dense hydrophobic film within the pores and interfacial regions of the material [22]. Lv et al. [23] demonstrated that organosilicon resins can uniformly coat aggregate surfaces, a finding substantiated by elemental distribution maps obtained through electron probe microanalysis. Additionally, boiling water tests revealed that aggregates coated with organosilicon resin exhibited a greater than 40% improvement in moisture isolation performance. The organosilicon film exhibits strong hydrophobicity and oleophobicity, reducing surface energy and inhibiting the penetration of moisture and organic media. Moreover, it enhances the overall integrity and stability of the interfacial structure to a certain extent [24]. However, the formation of this film on the asphalt pavement surface slightly reduces the anti-skid performance of the roadway.

Anti-skid performance is a fundamental functional attribute that must be consistently maintained throughout the entire lifecycle of an asphalt pavement [25,26]. This performance is intrinsically linked to the structural morphology and void distribution of the pavement surface layer, alongside the mineral hardness, surface roughness, and angularity of the aggregates [27,28,29]. During actual service, however, the synergistic effects of traffic abrasion, rutting deformation, and aggregate polishing progressively degrade the micro-textural roughness. This process ablates the asphalt mastic film, leaving the exposed mineral aggregate surfaces increasingly smooth. To mitigate the attenuation of anti-skid performance induced by organosilicon film formation on the pavement surface, Guo et al. [22] incorporated anti-skid aggregates to enhance surface friction. Nevertheless, these aggregates proved highly susceptible to stripping under vehicular friction and wheel tracking, thereby failing to maintain long-term durability. Consequently, the development of functional wearing course structures capable of withstanding complex service environments has emerged as a critical research frontier in contemporary asphalt pavement engineering [30].

**Figure 1:**
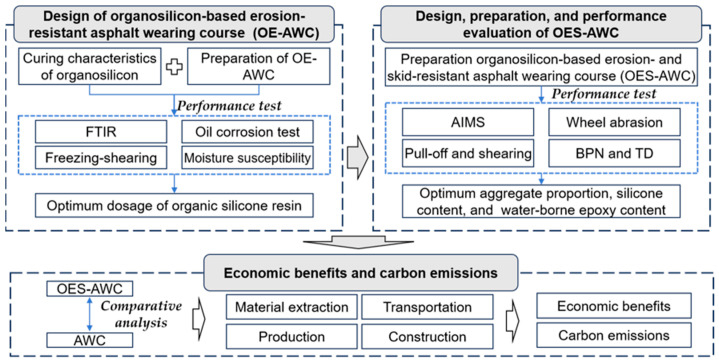
The research roadmap.

To address these challenges, this study develops an organosilicon-based asphalt wearing course that integrates environmental erosion resistance with long-term skid performance. The proposed organosilicon-based erosion- and skid-resistant asphalt wearing course (OES-AWC) consists of an erosion-resistant asphalt concrete matrix and a durable anti-skid surface layer. Compared to conventional AWC, the OES-AWC simplifies the construction process and reduces associated carbon emissions. Furthermore, in contrast to traditional organosilicon resin fog sealants, the OES-AWC provides better skid resistance while maintaining the internal interfacial protection necessary for long-term durability. In this system, organosilicon treatment is employed to construct a hydrophobic protective network for mitigating moisture damage, fuel erosion, and ice adhesion, while the anti-skid surface layer is optimized to maintain surface texture and friction stability under traffic abrasion. Waterborne epoxy resin is further introduced to enhance aggregate anchorage and improve the durability of the surface layer. The curing behavior and protective mechanism of organosilicon were characterized by FTIR, moisture-induced sensitivity, oil erosion, and freezing-shearing tests. The surface-layer design was optimized through aggregate morphology analysis, abrasion testing, pull-off and interfacial shearing tests, British pendulum testing, and texture depth measurements. The rutting resistance, stripping durability, long-term skid performance, and life-cycle environmental and economic benefits of the integrated OES-AWC were then systematically evaluated. The technical roadmap of this study is illustrated in Figure 1.

First, the curing characteristics of the organosilicon were investigated; through moisture-induced sensitivity test, oil erosion tests, and freeze-shear tests, its protective mechanisms for the asphalt matrix and the optimal dosage were elucidated. Subsequently, emery was selected as the optimal anti-skid aggregate. By regulating the binder-to-aggregate ratio (ROA) and the organosilicon content, the abrasion resistance and anti-skid performance of the wearing course were optimized, and the underlying mechanisms were revealed. Then, a waterborne epoxy resin (WER) was introduced to enhance the overall durability of the anti-skid system. The effectiveness of the composite-modified wearing course in resisting rutting and stripping, alongside its capacity to maintain long-term anti-skid performance, was comprehensively evaluated through wet track abrasion testing, wheel tracking tests, and prolonged anti-skid assessments. Finally, a comparative life cycle assessment was conducted to evaluate the environmental and economic benefits of OES-AWC.

## 2. Materials and Experimental Methods

### 2.1. Raw Materials

The AC-13 asphalt wearing course was prepared using SBS-modified asphalt with an asphalt–aggregate ratio of 5.0% and an air void content of 4.2%. An organosilicon material from Hubei, China, was applied to enhance anti-erosion performance. The material exhibited a dynamic viscosity of 24.8 mPa·s at ambient temperature. Basalt and emery aggregates (0.60–1.18 mm) were selected as anti-skid materials and bonded using waterborne epoxy resin. The key technical properties of the SBS-modified asphalt, aggregates and epoxy system are summarized in Table 1, Table 2 and Table 3.

### 2.2. Performance Test of Raw Materials

#### 2.2.1. Curing Characteristics of Organosilicon and Waterborne Epoxy Resin

The reactive oxygen-containing groups within the liquid organosilicon resin react with atmospheric moisture to generate silanol (Si-OH) groups. The subsequent polycondensation of these silanol groups forms a cross-linked siloxane (-Si-O-Si-) network, thereby imparting both adhesive and mechanical strength to the material. Fourier transform infrared (FTIR) spectroscopy (Nicolet iS50 FTIR, Shanghai, China) was employed to monitor the relative content and conversion rate (CR) of the Si-OH functional groups within the organosilicon. By calculating these parameters according to Equations (1) and (2), the progression of the curing process could be quantitatively evaluated.

(1) $$Content=S_{Si\text{-}OH}/S_{500\text{–}4000}$$

(2) $${CR}_{t}=1-\frac{{Content}_{t}}{{Content}_{0}}$$

where $S_{Si\text{-}OH}$ and $S_{500\text{–}4000}$ represent the absorption peak area of the Si-OH functional group and the total absorption peak area within the 500–4000 cm^−1^ range; ${CR}_{t}$ denotes the conversion rate of the Si-OH groups at curing time t; ${Content}_{t}$ and ${Content}_{0}$ indicate the relative Si-OH content at time t and in the initial uncured organosilicon, respectively.

Furthermore, the micro-morphological evolution of the waterborne epoxy emulsion during the water evaporation process—specifically encompassing demulsification, phase inversion, and subsequent film formation—was systematically observed using a BXF-150 epifluorescence microscope (Shanghai Bingyu Optical Instrument Co., Ltd., Shanghai, China).

#### 2.2.2. Particle Characterization of Aggregate

**Figure 2:**
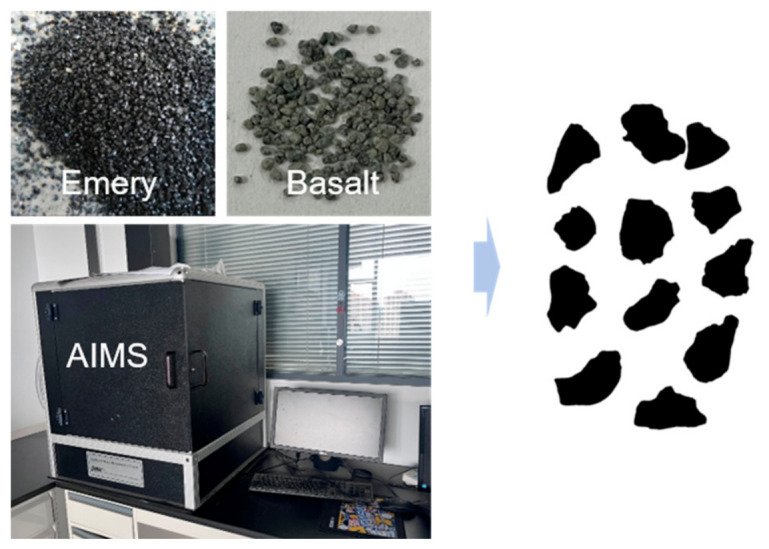
Particle characterization of anti-skid aggregates.

The characterization process for the morphological properties of the aggregates is illustrated in Figure 2. Initially, the two-dimensional morphologies of the emery and basalt aggregates were acquired utilizing an aggregate image measurement system (AIMS) (Pine Instrument Company, Grove City, PA, USA), as depicted in Figure 2. Subsequently, the micro-textural features of these aggregates were quantitatively evaluated by calculating their two-dimensional form index (F_2D_) and geometric anisotropy (G_A_) index [22].

### 2.3. Preparation and Performance Evaluation of Organosilicon-Based Erosion-Resistant Asphalt Wearing Course (OE-AWC)

#### 2.3.1. Preparation of OE-AWC

**Figure 3:**
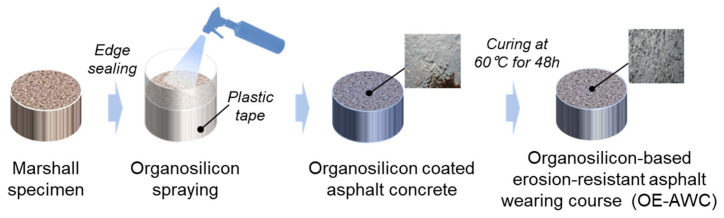
Preparation of organosilicon-based erosion-resistant asphalt wearing course.

Figure 3 illustrates the fabrication process of the organosilicon-based erosion-resistant asphalt wearing course (OE-AWC). The liquid organosilicon was uniformly sprayed onto the surface of the asphalt concrete at application rates of 200, 400, and 600 g/m^2^. Subsequently, the organosilicon-coated specimens were cured at 60 °C for 48 h to yield the final OE-AWC.

#### 2.3.2. Moisture-Induced Sensitivity Teste

**Figure 4:**
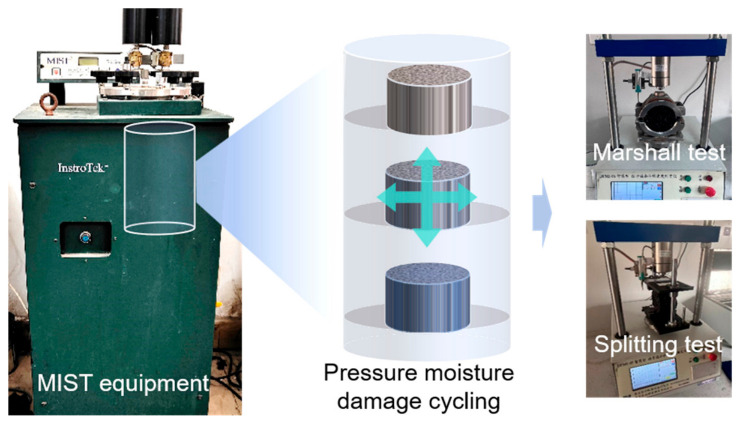
Hydrodynamic damage test.

The moisture-induced sensitivity tester (InstroTek, Inc., Raleigh, NC, USA) (Figure 4) was employed to simulate the hydrodynamic scouring action that asphalt concrete undergoes in actual service environments by applying elevated temperatures and cyclic dynamic pressure. The moisture damage resistance of the OE-AWC was evaluated by comparing the Marshall stability and splitting strength of the specimens before and after the simulated moisture conditioning (60 °C, 414 kPa, and 3500 cycles). Subsequently, the retained Marshall stability (RMS) and the retained splitting strength ratio were calculated utilizing Equations (3) and (4), respectively. Both the Marshall stability and indirect tensile strength were tested 4 times, and the average value with standard deviation was recorded.

(3) $${RMS}_{M}=\frac{{MS}_{M}}{{MS}_{0}}\times 100\%$$

(4) $${TSR}_{M}=\frac{{SP}_{M}}{{SP}_{0}}\times 100\%$$

where ${MS}_{0}$ and ${MS}_{M}$ respectively represent Marshall stability before and after hydrodynamic damage, and ${SP}_{0}$ and ${SP}_{M}$ respectively represent splitting strength before and after hydrodynamic damage.

#### 2.3.3. Oil Corrosion Test

**Figure 5:**
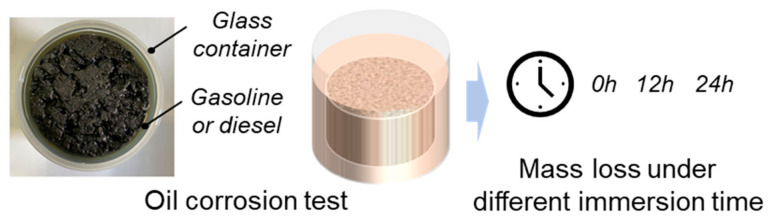
Oil corrosion test.

In specialized sections such as port roadways and entrances to large-scale construction sites, asphalt pavements are highly susceptible to fuel erosion, which precipitates binder dissolution and aggregate stripping. To evaluate the impact of the organosilicon treatment on the oil erosion resistance of the asphalt concrete, the mass loss of the specimens was measured following immersion in fuels (gasoline and diesel) for varying durations (12 and 24 h), as illustrated in Figure 5. The fuel immersion process was conducted at 25 °C. Subsequently, the specimens were removed and allowed to stand for 24 h prior to weighing to determine their post-erosion mass.

#### 2.3.4. Freezing-Shearing Test

**Figure 6:**
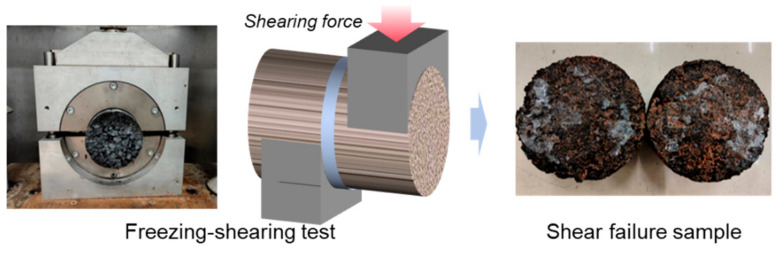
Freezing–shearing test.

A freezing-shearing test (Figure 6) was employed to assess the effect of the organosilicon treatment on the anti-icing performance of the asphalt concrete. Two standard Marshall specimens were vertically stacked, and water was introduced into the interfacial gap between them. The assembly was subsequently subjected to a freezing environment at −15 °C for 4 h, facilitating the formation of an ice layer that rigidly bonded the specimens together. Following this conditioning period, a shear test was conducted on the ice-bonded specimens utilizing a Universal Testing Machine UTM-25 (IPC Global Pty Ltd., Boronia, VIC, Australia) at a controlled temperature of −15 °C and a constant loading rate of 1 mm/min. The maximum shear force required to induce failure at the interface was recorded to quantitatively evaluate the anti-icing performance of the organosilicon-based asphalt concrete. The maximum shear force was tested 6 times, and the average value with standard deviation was recorded.

#### 2.3.5. Pull-Off and Interfacial Shearing Test

**Figure 7:**
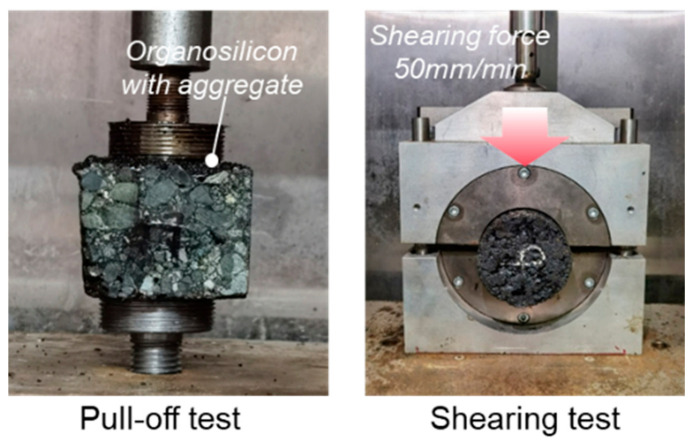
Pull-off and interfacial shearing test.

Pull-off and interfacial shearing tests (Figure 7) were conducted to evaluate the interlayer bonding performance of the asphalt concrete treated with the organosilicon-based sand fog seal. For the pull-off test, a treated asphalt concrete specimen was bonded at one end to a metal stub using the same fog seal material. The assembly was then mounted on a Universal Testing Machine (UTM-25, Australia) and subjected to a tensile load at a constant displacement rate of 50 mm/min until failure occurred at the bonding interface. The maximum tensile force was recorded.

For the interfacial shearing test, two standard Marshall specimens were bonded together horizontally using the sand fog seal to form a composite specimen. Subsequently, a shear test was performed on this composite specimen using the UTM-25, following a procedure similar to the Freezing-shearing test described in Section 2.3.4. The test was conducted at a constant loading rate of 50 mm/min. The maximum shear force required to cause interfacial failure was measured to assess the shear bonding strength.

### 2.4. Design, Preparation, and Performance Evaluation of OES-AWC

The organosilicon-based erosion- and skid-resistant asphalt wearing course (OES-AWC) is fundamentally composed of an erosion-resistant asphalt wearing course (OE-AWC) matrix and a skid-resistant sand-based fog seal. Section 2.3 details the performance evaluations of the OE-AWC, the results of which serve as the basis for determining the optimal organosilicon dosage. Subsequently, Section 2.4 outlines the design methodology for the skid-resistant sand seal, alongside the comprehensive performance testing protocols for the integrated OES-AWC system.

#### 2.4.1. Preparation of Skid-Resistant Fog Seal and OES-AWC

**Figure 8:**
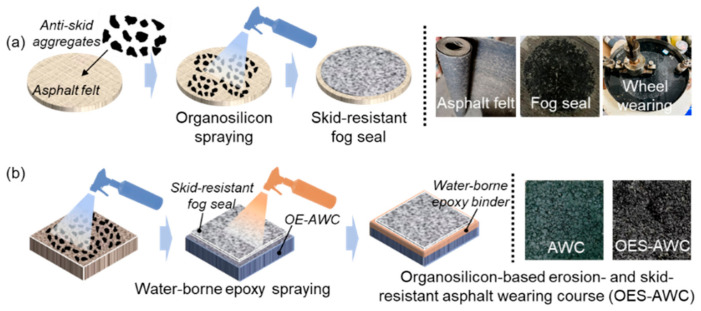
Preparation of (**a**) skid-resistant fog seal and (**b**) OES-AWC.

In this study, two distinct substrates—asphalt felt and wheel tracking specimens—were employed for the material proportioning design and the evaluation of pavement performance, respectively. Figure 8a,b depict the fabrication processes of the skid-resistant fog seal and the OES-AWC, respectively. During preparation, the materials were uniformly applied in a strict sequence: anti-skid aggregates, organosilicon, and waterborne epoxy resin. For the skid-resistant fog seal, the application rates of the organosilicon were specified as 400, 550, 700, 850, and 1000 g/m^2^. Additionally, the mass ratios of organosilicon to anti-skid aggregate (ROA) were systematically designed as 30:70, 30:65, 35:70, 35:65, 40:70, 40:65, 45:70, and 45:65.

#### 2.4.2. Skid Resistance and Durability Test

Table 4 summarizes the performance testing methodologies employed for both the skid-resistant fog seal and the OES-AWC. These protocols include the wet track abrasion test, the British pendulum test, the texture depth test, the wheel tracking test, and the water permeability test. Collectively, these tests were conducted to systematically evaluate the abrasion resistance, anti-skid properties, overall durability, and impermeability of the proposed pavement structures.

The wet track abrasion test and the wheel tracking test were conducted on the skid-resistant fog seal and the OES-AWC, respectively, to evaluate their abrasion resistance and structural durability. These performance metrics were assessed by quantifying the stripping rate of the anti-skid aggregates following the completion of the respective tests. The specific calculation methodologies are expressed in Equations (5) and (6).

(5) $$Mass\;loss\;ratio=\frac{m_{a0}-m_{ai}}{m_{a0}}\times 100\%$$

(6) $$Spalling\;rate=\frac{m_{r0}-m_{ri}}{m_{r0}}\times 100\%$$

where $m_{a0}$ and $m_{ai}$ represent the mass of skid-resistant fog seal before and after wheel wearing, $m_{r0}$ and $m_{ri}$ denote the mass of OES-AWC before and after wheel tracking.

## 3. Organosilicon Property and Its Influence on Asphalt Wearing Course

### 3.1. Curing Characteristics of Organosilicon

**Figure 9:**
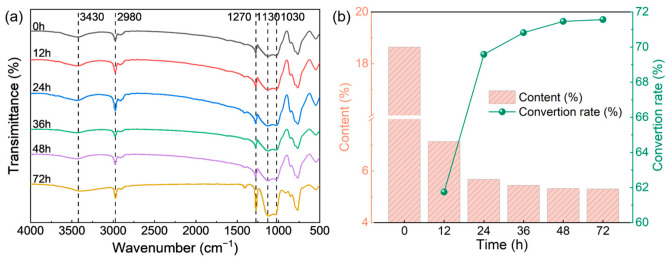
Curing characteristics of organosilicon: (**a**) infrared spectrum; (**b**) conversion rate.

The (CH_3_CH_2_O)_3_-Si-R groups within the organosilicon resin undergo hydrolysis to form silanol (Si-OH) groups, which manifest as a broad stretching vibration peak in the high-wavenumber region (3430 cm^−1^) of the infrared spectrum, as shown in Figure 9a. Subsequent polycondensation between the generated R-Si-(OH)_3_ groups leads to the formation of siloxane (Si-O-Si) bonds, eventually evolving into a robust -(Si-O-Si)_n_- three-dimensional network as the reaction progresses. In Figure 9a, the stretching vibrations of the Si-O-Si bonds yield distinct absorption peaks at 1130 cm^−1^ and 1030 cm^−1^, while the stretching and bending vibrations of the alkyl groups are identified at 2980 cm^−1^ and 1270 cm^−1^, respectively.

To evaluate the curing kinetics of the organosilicon, the relative content of Si-OH groups was monitored over time, as presented in Figure 9b. The curing process can be clearly categorized into three distinct stages: the acceleration phase (0–24 h), the deceleration phase (24–36 h), and the plateau phase (36–72 h). During the acceleration phase, particularly within the first 12 h, the relative Si-OH content dropped precipitously from 18.6% to 7.1%, achieving a conversion rate of 61.8%. This indicates the rapid establishment of the cross-linked network. In the deceleration phase, the Si-OH content further decreased to 5.4%, where the reaction rate was significantly hindered by diffusion-controlled mechanisms. Finally, the Si-OH content stabilized at approximately 5.3%, signaling the transition into the plateau phase. This indicates that the primary network has effectively formed and the reaction of residual functional groups has reached a near-stagnant state. These three-stage kinetic characteristics encapsulate the complete structural transformation of the organosilicon from a solution to a three-dimensional network. The acceleration phase corresponds to rapid early film formation, which is conducive to the swift development of moisture resistance post-construction, while the deceleration and plateau phases ensure the long-term chemical stability and durability of the coating.

### 3.2. Dynamic Moisture Damage-Resistance of OE-AWC

**Figure 10:**
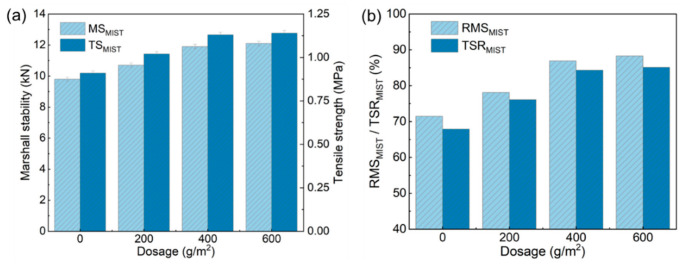
Dynamic moisture damage-resistance of OE-AWC: (**a**) Marshall stability and tensile strength after MIST; (**b**) RMS and TSR at different organosilicon content.

The effect of organosilicon treatment on the moisture damage resistance of asphalt mixtures was assessed using the moisture induced stress tester (MIST), with performance quantified by the retained Marshall stability (RMS) and tensile strength ratio (TSR). As shown in Figure 10, both RMS and TSR exhibited a significant initial increase followed by stabilization as the application rate rose from 0 to 600 g/m^2^. The untreated control showed the lowest resistance, with RMS and TSR at 71.5% and 67.9%, corresponding to post-MIST Marshall stability and tensile strength dropping to 9.8 kN and 0.91 MPa from initial values of 13.7 kN and 1.34 MPa. At 400 g/m^2^, the RMS and TSR reached 86.9% and 84.3%, representing increases of approximately 21.4% and 24.2% over the control. This improvement is mainly attributed to the organosilicon resin forming a continuous, hydrophobic protective film that shields the asphalt-aggregate interface from moisture intrusion. However, further increasing the dosage to 600 g/m^2^ yielded marginal gains, which indicates that a complete protective barrier might already be established at 400 g/m^2^, and additional organosilicon does not significantly enhance interfacial protection.

### 3.3. Oil Corrosion-Resistance of OE-AWC

**Figure 11:**
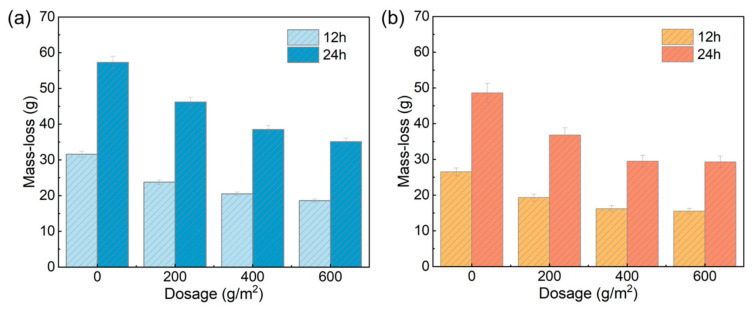
The mass-loss of OE-AWC after oil erosion: (**a**) gasoline; (**b**) diesel.

The gasoline and diesel erosion resistance of the organosilicon-based asphalt mixtures are presented in Figure 11a,b, respectively. The results show that organosilicon treatment significantly improves fuel erosion resistance, with the protective efficiency dependent on fuel solvency, resin dosage, and immersion duration. Gasoline, rich in light aromatics and short-chain alkanes, exhibits a stronger ability to dissolve and strip the asphalt binder than diesel, resulting in greater mass loss under identical conditions. As the organosilicon dosage increased, the mass loss for both fuels decreased initially and then stabilized. The optimal performance was achieved at 400 g/m^2^, where gasoline-induced mass loss was reduced by 35.1% and 32.8% after 12 and 24 h, respectively, while diesel-induced reductions reached 38.9% and 39.3%. Further increasing the dosage to 600 g/m^2^ provided negligible improvement, indicating that near-complete interfacial coverage is already achieved at 400 g/m^2^, beyond which additional resin does not significantly enhance the protective barrier.

### 3.4. Anti-Icing Performance of OE-AWC

**Figure 12:**
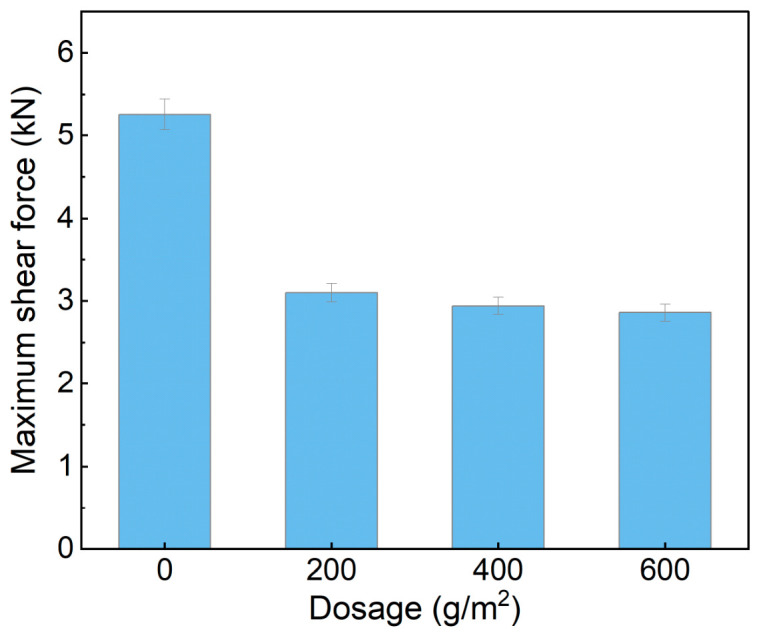
The anti-icing performance of OE-AWC.

Figure 12 presents the results of the freezing–shearing tests on organosilicon-treated asphalt mixtures. The incorporation of organosilicon significantly reduced the bonding strength at the asphalt–ice interface, exhibiting a clear threshold effect. The untreated specimens showed a high shear force of 5.26 kN, attributed to the high surface energy of asphalt that promotes strong physico-chemical bonding with ice. With the application of 200 g/m^2^ organosilicon, the shear force decreased sharply to 3.10 kN. This reduction is mainly due to the formation of a hydrophobic -(Si-O-Si)_n_- network, which lowers interfacial surface energy and shifts the failure mode from interfacial stripping to cohesive failure within the ice or interfacial slippage. Further increasing the dosage to 400 and 600 g/m^2^ resulted in only slight reductions to 2.94 kN and 2.86 kN, respectively. This limited improvement indicates that effective surface coverage is already achieved at low dosage, while higher dosages mainly increase coating thickness or penetration depth with minimal additional impact on surface energy. Consequently, the anti-icing performance reaches a plateau beyond the initial application threshold.

## 4. Design and Performance Test of Skid-Resistant Asphalt Wearing Course

### 4.1. Particle Characteristics of Anti-Skid Aggregate

**Figure 13:**
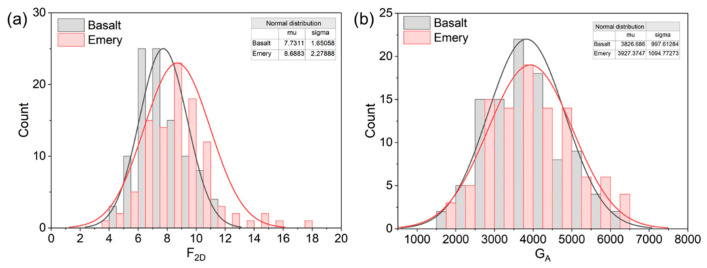
Particle characteristics of anti-skid aggregate: (**a**) F_2D_; (**b**) G_A_.

Figure 13a,b illustrate the distribution characteristics of the two-dimensional form index (F_2D_) and the geometric anisotropy (G_A_) index for the basalt and emery aggregates, respectively. As shown in the histogram in Figure 13a, the F_2D_ distribution for basalt is more concentrated, with a mean (μ) of 7.731 and a standard deviation (σ) of 1.651, peaking within the 6–8 range. This suggests a more regular two-dimensional morphology and highly consistent angularity. Conversely, the F_2D_ distribution for emery is more dispersed (μ = 8.689, σ = 2.279), with the peak shifting toward the 8–10 range. This indicates greater diversity in particle shapes, including a higher proportion of extremely flat or elongated particles. Such morphological differences suggest that while basalt is more conducive to forming a stable skeletal structure, the use of emery requires careful mix proportion optimization to mitigate the risk of stress concentration arising from shape variability. Regarding geometric anisotropy, as illustrated in Figure 13b, the mean G_A_ for emery (3927.37) is slightly higher than that of basalt (3826.69). Furthermore, the broader distribution of GA values for emery underscores its stronger geometric anisotropy and more pronounced morphological heterogeneity, which suggests a superior potential for enhancing anti-skid performance.

### 4.2. Influence of ROA on the Abrasion Resistance of OES-AWC

**Figure 14:**
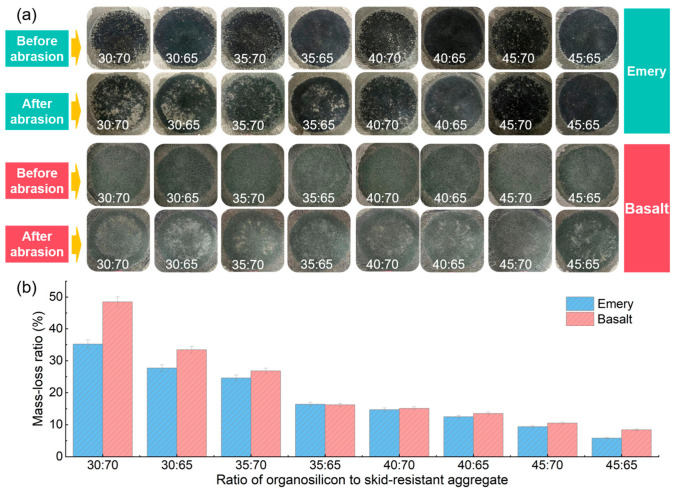
The abrasion resistance of OES-AWC: (**a**) appearance; (**b**) mass-loss ratio.

In the organosilicon-based erosion- and skid-resistant asphalt wearing course (OES-AWC), the organosilicon component provides the asphalt concrete matrix with enhanced anti-erosion capabilities due to its intrinsic hydrophobic and oleophobic properties. Conversely, the anti-skid performance of the OES-AWC is primarily derived from the surface wearing course, which is composed of anti-skid aggregates and a binder. Based on preliminary experimental results, the organosilicon dosage for the anti-skid wearing course was fixed at 400 g/m^2^, while the optimal binder-to-aggregate ratio (ROA) was determined through abrasion testing. Figure 14a,b illustrate the results of the wet track abrasion tests for anti-skid wearing courses with varying ROA values and aggregate types. As shown in Figure 14a, the specimens across different ROA and aggregate groups initially exhibited a uniform coverage of the wearing course. However, the emery group demonstrated more significant textural prominence due to the higher hardness and irregular morphology of the aggregates. In contrast, the basalt group presented a relatively smoother surface owing to the uniform shape and compact distribution of the aggregates. Following the abrasion process, both specimen groups exhibited varying degrees of surface wear and degradation.

Figure 14b provides a quantitative analysis of the mass loss rate of the wearing course, a parameter that directly reflects its resistance to wear-induced stripping. A lower mass loss rate indicates superior material retention and enhanced abrasion durability. At identical binder-to-aggregate ratios (ROA), the mass loss rates for the emery specimens were significantly lower than those for the basalt specimens. At an ROA of 30:70, the mass loss rate for emery was 35.2%, whereas that of basalt reached 48.5%. This difference is attributed to the higher hardness of emery, which enhances resistance to wear and stripping. Furthermore, increasing the proportion of the binder markedly reduced the mass loss for both aggregate types. Specifically, for the emery group, the mass loss rate plummeted from 35.2% at an ROA of 30:70 to 5.8% at an ROA of 45:65—a reduction exceeding 83%. This underscores the critical role of an adequate binder content in effectively encapsulating and anchoring the high-hardness aggregates to form a coherent, integrated wearing course.

### 4.3. Influence of Organosilicon Content on the Adhesive Performance of OES-AWC

**Figure 15:**
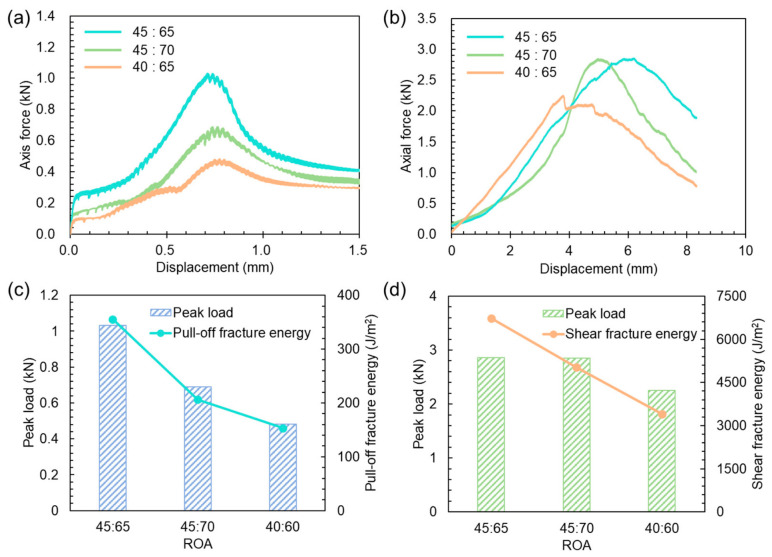
The adhesive performance of OES-AWC: (**a**) pull-off force; (**b**) interfacial shear force; (**c**) pull-off fracture energy; (**d**) shear fracture energy.

Figure 15 illustrates the interfacial bonding performance of organosilicon-based sand fog seal under different ROA ratios. Figure 15a,b present the force-displacement curves of various specimens obtained from the pull-off and shear tests, respectively. As shown in Figure 15a, the interfacial force of all specimens increases rapidly with increasing pull-off displacement. The specimen with an ROA ratio of 45:65 exhibits the fastest increase in force, as well as the highest pull-off strength and fracture energy, reaching 1.03 kN and 355.7 J/m^2^, respectively, as shown in Figure 15c, which are nearly twice those of the specimen with an ROA ratio of 40:60. This improvement is primarily attributed to the increased organosilicon content, which enhances interfacial bonding performance.

Figure 15b,d show the force evolution and fracture energy of the specimens in the shear test, respectively, with trends generally consistent with those observed in the tensile test. The specimens with ROA ratios of 45:65 and 45:70 exhibit similar peak shear forces; however, the former corresponds to a larger displacement at peak load, resulting in a higher fracture energy of 6735.1 J/m^2^, which is 34.0% greater than that of the latter. In addition, as shown in Figure 15b, the shear force-displacement curves of the three specimens display significant differences, reflecting distinct interfacial bonding and failure mechanisms. The specimen with an ROA ratio of 45:65 exhibits the highest peak (2.86 kN) and a gradual, stepwise post-peak decline, with the largest enclosed area under the curve, indicating substantial energy dissipation through sustained friction and sliding during shearing, and thus superior toughness. In contrast, the specimen with an ROA ratio of 45:70 shows a more rapid post-peak decline and a reduced curve area, suggesting diminished toughness. The specimen with an ROA ratio of 40:65 exhibits the lowest peak force (2.25 kN) and a sharp drop after reaching the peak, resulting in the steepest curve, which indicates a more brittle interfacial failure with the weakest energy dissipation capacity; its fracture energy is only 3408.6 J/m^2^.

These differences are mainly attributed to the influence of the ROA ratio on the integrity of organosilicon coating on aggregate surfaces and the toughness of the cured film. When the organosilicon content is insufficient, the resulting bonding film is thin and discontinuous, leading to increased interfacial brittleness and poor deformation compatibility. In contrast, the optimal ROA ratio of 45:65 ensures the formation of a strong and continuous bonding film on the aggregate surface, enabling effective stress transfer and promoting more extensive energy dissipation under shear loading.

### 4.4. Influence of Organosilicon Content on the Skid Resistance of OES-AWC

**Figure 16:**
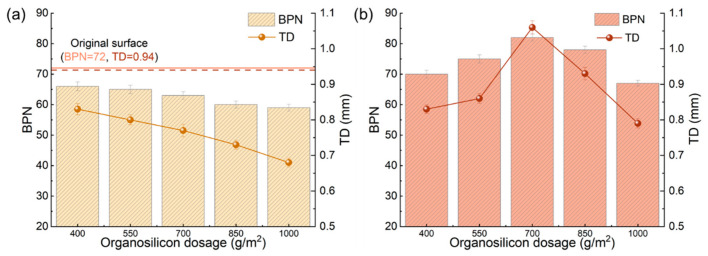
The skid resistance of AWC: (**a**) without skid-resistant aggregate; (**b**) OES-AWC.

Based on the optimized ROA of 45:65, the influence of organosilicon content on the performance of the anti-skid wearing course was further investigated using the British pendulum number (BPN) and texture depth (TD) as indicators. Figure 16a,b illustrate the regulatory effects of organosilicon application rates on the performance of conventional AWC and OE-AWC, respectively. As shown in Figure 16a for the conventional AWC (without anti-skid aggregates), both BPN and TD exhibited a monotonic decline as the organosilicon content increased from 400 to 1000 g/m^2^, dropping from 66 to 59 and from 0.83 mm to 0.68 mm, respectively. This degradation is primarily attributed to the formation of a smooth organosilicon film on the asphalt surface, which reduces frictional resistance, while the cured resin fills the inherent surface voids, thereby diminishing the texture depth.

In contrast, the anti-skid performance of the OE-AWC followed a non-monotonic trend of initial enhancement followed by a subsequent decrease, as depicted in Figure 16b. When the application rate of organosilicon increased from 400 to 700 g/m^2^, the BPN and TD simultaneously improved, reaching peak values of 82 and 1.06 mm, respectively. However, as the content further increased to 1000 g/m^2^, both indicators reversed, falling to 67 and 0.79 mm. This behavior is governed by the balance between aggregate anchoring and excessive binder filling. Once the application rate exceeds a critical threshold (700 g/m^2^), the excess binder overfills the voids between the aggregates, leading to excessive densification. This effectively masks the rough texture of the aggregates and reduces the macroscopic texture depth, thereby compromising the effective tire-pavement engagement. Therefore, the dosage of 700 g/m^2^ is suitable from the perspective of skid resistance performance.

### 4.5. Influence of Organosilicon Content on the Durability of OES-AWC

**Figure 17:**
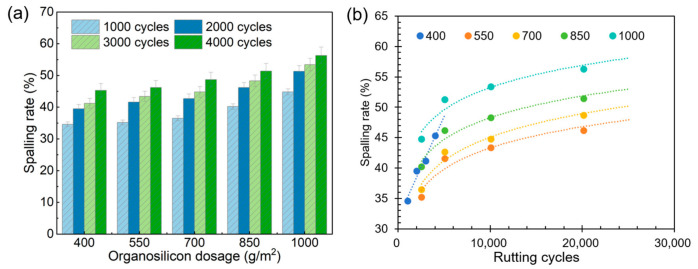
The durability of OES-AWC under different rutting cycles: (**a**) spalling rate at different organosilicon content; (**b**) fitting curve between spalling rate and rutting cycle.

Beyond mass loss from abrasion, the stripping induced by wheel tracking loads is a critical factor that directly governs the long-term durability of the wearing course. Figure 17a illustrates the evolution of the aggregate stripping rate in OES-AWC as a function of organosilicon content and wheel tracking cycles. Overall, the stripping rate for all organosilicon dosage groups exhibited an upward trend as the number of loading cycles increased from 1000 to 4000. Under a fixed ROA, an increase in organosilicon content leads to a proportional rise in the absolute volume of anti-skid aggregates, resulting in a continuous increase in the physical thickness of the wearing course. Although the organosilicon resin forms a hydrophobic film that enhances interfacial resistance to moisture, its cohesive strength proves insufficient to fully constrain the relative displacement of large aggregate volumes when subjected to the repetitive high-frequency shear stress of wheel tracking. Particularly during the development of rutting, the tendency for interlayer slippage, exacerbated by the increased layer thickness, is amplified, leading to consistently high stripping rates. At 4000 cycles, the stripping rate reached 45.3% for the 400 g/m^2^ group and 56.3% for the 1000 g/m^2^ group. These results indicate that relying solely on the organosilicon surface film is insufficient to withstand the mechanical spalling induced by permanent deformation (rutting) under high-temperature and heavy-traffic conditions. Furthermore, curve fitting results of the spalling rate are shown in Figure 17b, and the fitting parameters are shown in Table 5.

When the organosilicon dosage is relatively low (400 g/m^2^), the spalling rate exhibits a linear relationship with the number of loading cycles, indicating that the spalling process is dominated by continuous and stable shear-induced damage. However, when the dosage increases to 550 g/m^2^ and above, the evolution of the spalling rate follows a logarithmic growth pattern. This transition from a linear to a logarithmic mode suggests that a higher organosilicon dosage can buffer stress and delay spalling at the initial stage, as evidenced by the significantly reduced slope of the fitted curve during the early rutting phase. At 1000 cycles, the fitted slope for the specimen with an organosilicon dosage of 700 g/m^2^ is 0.0056, representing an 83.5% reduction compared to 0.034 for the 400 g/m^2^ specimen. Nevertheless, as the loading cycles proceed, the inherent limitation of insufficient cohesive strength in organosilicon becomes increasingly pronounced, and the spalling rate gradually enters a plateau of slow growth at a relatively high level. For the high-dosage group (1000 g/m^2^), the spalling rate remains as high as 56.3% after 4000 cycles, and the relatively large constant term in the fitted function (4.6049) indicates a high initial risk of spalling. These results further demonstrate that simply increasing the organosilicon dosage can alter the damage accumulation pattern but cannot overcome its intrinsic mechanical strength limitations. Therefore, the incorporation of waterborne epoxy resin as a reinforcing phase is necessary to stabilize the anti-skid aggregates and enhance the overall durability of the wearing course.

### 4.6. Curing Characteristics of Waterborne Epoxy and Its Enhancement Effect of OES-AWC

#### 4.6.1. Curing Characteristics of Waterborne Epoxy

**Figure 18:**
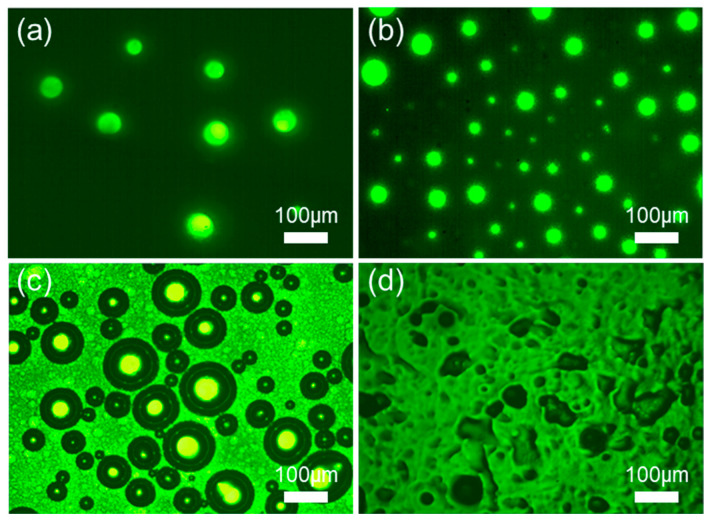
Fluorescence micrograph (magnification of 40×) of waterborne epoxy resin: (**a**) 0 min; (**b**) 3 min; (**c**) 6 min; (**d**) 10 min.

Waterborne epoxy resin, an epoxy-based material utilizing water as the dispersive medium, possesses high-strength characteristics upon demulsification and curing, thereby demonstrating significant potential for inhibiting aggregate stripping in anti-skid wearing courses. Figure 18 presents the dynamic demulsification-curing process of the waterborne epoxy resin through fluorescence microscopy (40×), providing a visual elucidation of its strength development mechanism. As shown in Figure 18a, the initial waterborne epoxy system exists as an oil-in-water (O/W) emulsion, where epoxy particles appear as discrete fluorescent bright spots stably dispersed within the aqueous phase. With the evaporation of water, the proximity between polymer particles increases, thereby enhancing the probability of inter-particle collisions; however, the system maintains its O/W morphology at this stage, as illustrated in Figure 18b. Subsequently, in Figure 18c, the primary and secondary amines within the curing agent react with the epoxy groups, initiating particle aggregation. As the majority of the water evaporates, the emulsion undergoes a phase inversion from an O/W type to a water-in-oil (W/O) type. In this transitional state, the epoxy resin becomes the continuous phase while water serves as the dispersed phase, allowing the polymer particles to begin coalescence. Once water evaporation and the curing reaction are substantially complete, a continuous resinous skeletal phase is established, which provides the necessary load-bearing capacity, as depicted in Figure 18d.

#### 4.6.2. Durability of OES-AWC Reinforced by Waterborne Epoxy

**Figure 19:**
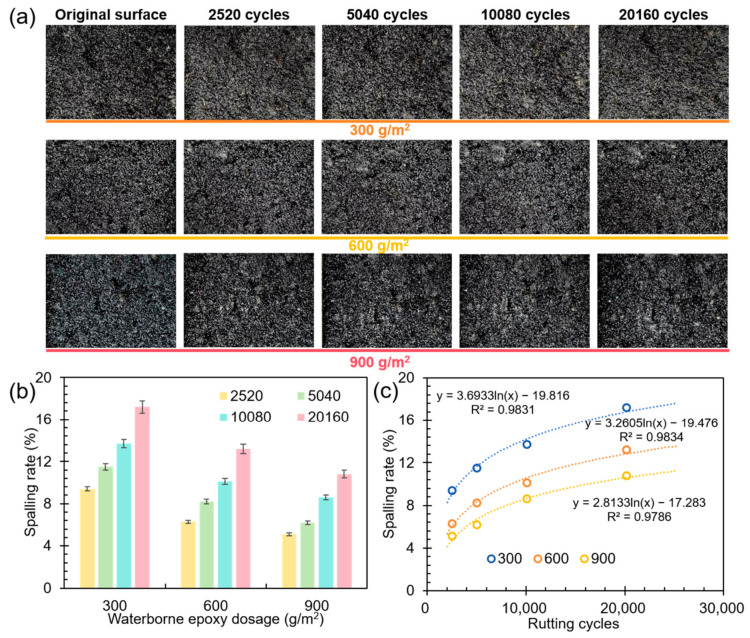
Aggregate spalling characteristics of OES-AWC under different rutting cycles: (**a**) macro-morphology; (**b**) spalling rate; (**c**) fitting curve.

The organosilicon-based anti-skid wearing course was reinforced with waterborne epoxy resin and applied to the asphalt pavement surface to yield the final organosilicon-based erosion- and skid-resistant asphalt wearing course (OES-AWC). The rutting durability of the OES-AWC was evaluated by measuring the anti-skid aggregate stripping rates across varying wheel tracking cycles, as illustrated in Figure 19. Figure 19a depicts the macroscopic morphological evolution of the OES-AWC under wheel tracking loads for different epoxy application rates, while Figure 19b quantitatively presents the corresponding stripping rates. Overall, the aggregate stripping rates of the OES-AWC remained below 12% after 5000 cycles, representing a marked improvement over the specimens without waterborne epoxy reinforcement (Figure 16), where stripping rates exceeded 30%.

The quantitative data and the fitting curve in Figure 19b,c clearly reveal the regulatory influence of the waterborne epoxy resin dosage on the rutting durability of the OES-AWC. At 2520 cycles, representing the early service stage, the stripping rates for the 300, 600, and 900 g/m^2^ groups were 9.4%, 6.3%, and 5.1%, respectively, exhibiting a monotonic decrease with increasing epoxy content. This demonstrates that the reinforcing effect of the epoxy is manifested as a significant reduction in the initial aggregate stripping rate. By 5040 cycles, the stripping rates for these three groups reached 11.5%, 7.6%, and 6.2%, respectively. Notably, the growth increment of the stripping rate narrowed as the epoxy content increased, suggesting that higher dosages more effectively decelerate the progression of aggregate loss.

Figure 19c illustrates that the evolution of aggregate spalling rate with rutting cycles under different epoxy resin dosages follows a logarithmic growth model. Although the spalling rate increases with the number of loading cycles, its growth rate gradually decreases and remains significantly lower than that of the system without epoxy resin. This behavior is primarily attributed to the continuous cured film and three-dimensional cross-linked network formed by the epoxy resin (Figure 18), which effectively dissipate and redistribute the shear stresses induced by wheel loading, thereby transforming aggregate loss from a rapid linear process into a slow and progressive one.

A quantitative assessment of the effect of epoxy resin dosage can be obtained by comparing the fitted equation parameters. In the logarithmic model y=a⋅ln(x)+b, the coefficient a represents the sensitivity of the spalling rate to loading cycles. Its value decreases from 3.6933 for the 300 g/m^2^ group to 2.8133 for the 900 g/m^2^ group, indicating that increasing the epoxy resin dosage significantly reduces the rate of damage accumulation. As a result, the high-dosage group is able to maintain the spalling rate below 15% even after 20,160 cycles, demonstrating excellent durability.

It should be noted that the successful field application of the waterborne epoxy-reinforced OES-AWC is contingent upon environmental conditions. The curing kinetics of the waterborne epoxy resin is highly sensitive to ambient humidity and temperature; therefore, construction should ideally occur under low-humidity conditions to facilitate proper film formation and cross-linking. Furthermore, given the time-dependent nature of strength development, a sufficient traffic closure period must be enforced to ensure the epoxy network achieves adequate cohesive strength prior to service loading. Future studies will explore accelerated curing agents to mitigate these field application constraints.

### 4.7. Long-Term Skid-Resistance of OES-AWC

**Figure 20:**
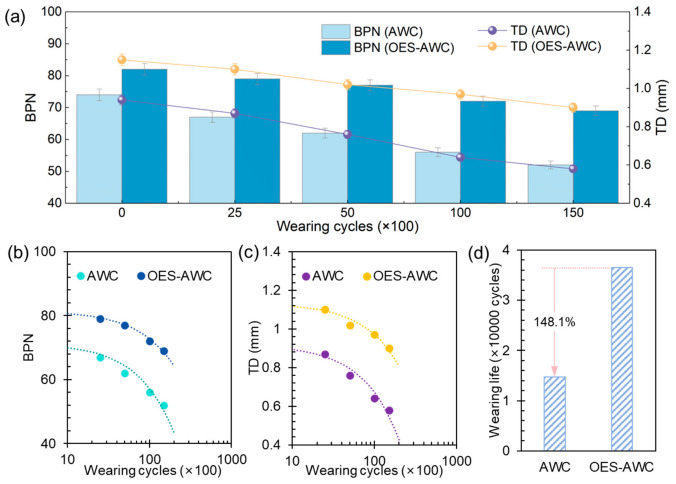
Skid-resistance of OES-AWC after different wearing cycles: (**a**) BPN and TD; (**b**) prediction model of BPN; (**c**) prediction model of TD; (**d**) wearing life.

Building upon the confirmed rutting durability, the anti-skid performance of the OES-AWC was further evaluated under various abrasion conditions to assess its long-term performance retention. Figure 20a illustrates the evolution of the BPN and texture depth (TD) for both the OES-AWC and conventional AWC. Throughout the 0–15,000 abrasion cycles, the BPN of the OES-AWC consistently remained higher than that of the conventional AWC, demonstrating more gradual degradation. Specifically, at the initial stage, the BPN of the OES-AWC reached 82, representing a 10.8% enhancement over the 74 recorded for the conventional AWC.

After 15,000 cycles abrasion, the OES-AWC maintained a BPN of 64, while the conventional AWC dropped to 52; the cumulative reduction for the former was only 15.9%, compared to 29.7% for the latter. Furthermore, the OES-AWC exhibited a significantly slower TD attenuation rate than the conventional AWC, ensuring robust mechanical engagement between the pavement surface and vehicle tires—a critical factor for sustaining long-term skid resistance. This superiority is attributed to the intrinsic skid resistance and surface roughness provided by the high-hardness emery aggregates, alongside the reinforcement from the waterborne epoxy resin. The continuous cured film formed by the resin effectively inhibits aggregate stripping, thereby preserving both the macro-texture and micro-texture of the surface over extended periods. Consequently, the OES-AWC demonstrates a clear advantage over conventional AWC in terms of long-term skid resistance retention and wear stability.

To further quantify the long-term skid resistance advantage of OES-AWC, the fitted relationships between abrasion cycles and both the BPN and TD are presented in Figure 20b,c, respectively, with the corresponding fitting coefficients listed in Table 6. The skid resistance of both AWC and OES-AWC exhibits a linear decay with increasing abrasion cycles; however, the decay rate of AWC is significantly higher than that of OES-AWC. Specifically, the slope of the fitted curve for AWC is 0.1407, which is 61.9% greater than that of OES-AWC. According to the *Specifications for Design of Highway Asphalt Pavement* [31], the minimum required texture depth is 0.55 mm. Based on the texture depth decay model, the abrasion life of AWC and OES-AWC was calculated, as shown in Figure 20d. The abrasion life of OES-AWC reaches 36,487 cycles, which is 148.1% higher than that of AWC. These results demonstrate that OES-AWC exhibits a pronounced advantage in long-term skid resistance retention and wear stability. This superior performance can be attributed to the high hardness and wear resistance of emery aggregates, as well as the effective suppression of aggregate spalling by the continuous cured film formed by the waterborne epoxy resin.

## 5. Life Cycle Environmental and Economic Analysis

### 5.1. Goal and Scope Definition

**Figure 21:**
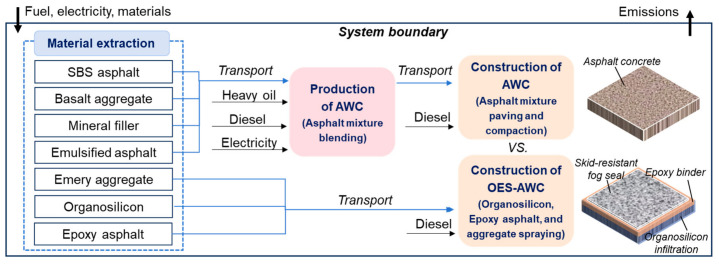
System boundary of life cycle assessment: cradle to gate.

The life cycle environmental and economic performance of OES-AWC and conventional AWC was evaluated in accordance with ISO 14040 and ISO 14044 standards [32,33]. The analysis focused on a typical asphalt pavement maintenance scenario with a length of 1 km, a width of 3.75 m, and a thickness of 0.02 m. The cradle-to-gate system boundary adopted in the LCA is illustrated in Figure 21. The conventional AWC consists of widely used SBS-modified asphalt concrete, with an asphalt-to-aggregate ratio of 5.0%. In contrast, the OES-AWC incorporates the material compositions determined in Section 3 and Section 4, including an organosilicon application rate of 7000 g/m^2^, a binder-to-aggregate ratio of 45:65, and an additional 600 g/m^2^ of waterborne epoxy resin.

### 5.2. Life Cycle Inventory, Impact Assessment, and Interpretation

The construction of OES-AWC and conventional AWC was carried out based on a full-scale engineering application, and the life cycle inventory data were obtained from both the literature and on-site investigations. Table 7, Table 8 and Table 9 present the unit carbon emissions, costs, and transportation distances of various energy sources and raw materials used in this study, as well as the material inputs required per unit pavement area and the energy consumption intensity of construction equipment. Based on this life cycle inventory, the carbon emissions and costs of OES-AWC and conventional AWC were quantified, which are widely recognized indicators for evaluating environmental and economic performance. The specific calculation methods, sensitivity analysis, and uncertainty analysis refer to the authors’ previous work [13,14].

### 5.3. Carbon Emissions and Cost of OES-AWC

**Figure 22:**
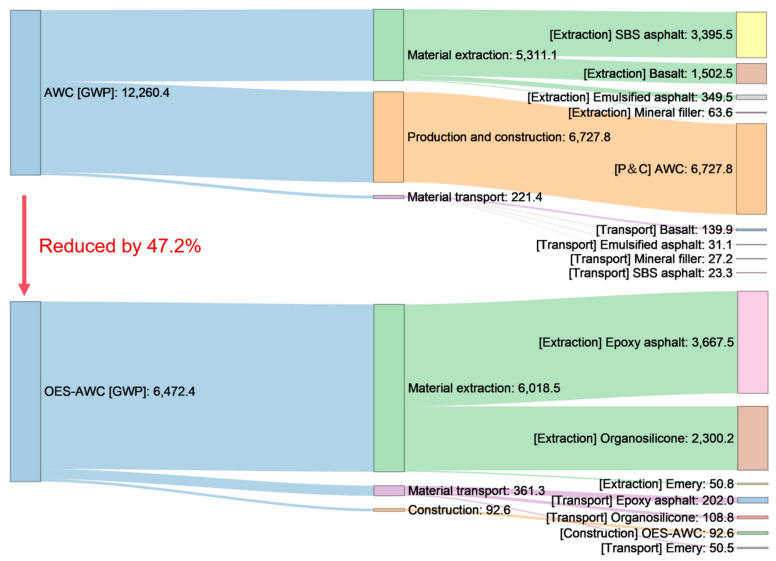
The carbon emission structure of OES-AWC and AWC.

The comparative life cycle assessment reveals the significant environmental advantages of OES-AWC, as illustrated in Figure 22. The cradle-to-gate equivalent CO_2_ emissions of the OES-AWC system are 6472.4 kg, representing a substantial reduction of 47.2% compared to 12,260.4 kg for the conventional AWC system. The carbon emission structure indicates that the dominant emission source for conventional AWC lies in the production and construction stages, contributing 54.9% of the total emissions. In contrast, the primary emission source for OES-AWC shifts to the material acquisition stage, accounting for 93.0% of the total emissions, mainly due to the extraction and processing of epoxy asphalt and organosilicon materials. Although these novel materials incur relatively higher initial carbon emission costs, the OES-AWC system completely eliminates the high-emission processes associated with conventional hot-mix asphalt production and SBS modification. Consequently, by fundamentally avoiding the most emission-intensive production stages inherent in traditional approaches, the OES-AWC strategy achieves a net reduction in carbon emissions.

**Figure 23:**
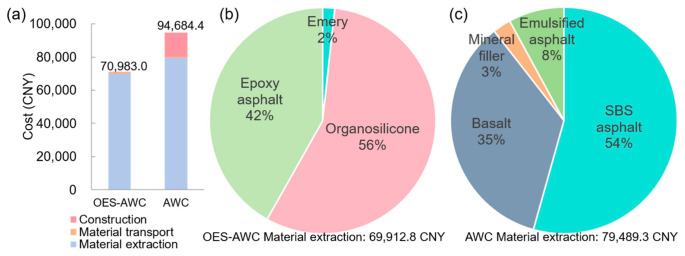
Cost structure of OES-AWC and AWC: (**a**) total cost; (**b**) material extraction of OES-AWC; (**c**) material extraction of AWC.

Figure 23 compares the cost structures of OES-AWC and conventional AWC. As shown in Figure 23a, the total cost of OES-AWC is 70,983.0 CNY, representing a 25.0% reduction compared to 94,684.4 CNY for conventional AWC. This cost saving is primarily attributed to the substantial reduction in construction and material transportation expenses, which is consistent with the previously observed optimization trend in carbon emission structure. Although raw material extraction dominates the cost composition of both OES-AWC and conventional AWC, their structural distributions differ significantly (Figure 23b,c). For OES-AWC, the raw material cost is mainly associated with organosilicon and epoxy asphalt. In contrast, SBS-modified asphalt accounts for the largest share of raw material cost in conventional AWC, reaching 54%, while basalt aggregates contribute a considerable 35%. This is largely due to the fact that aggregates typically constitute approximately 95% of the total mixture by mass. Therefore, by adopting a simplified and efficient material system, OES-AWC achieves comprehensive optimization in terms of material types, consumption, and associated construction and transportation costs, while maintaining performance, ultimately resulting in a lower overall life cycle construction cost.

### 5.4. Sensitivity and Uncertainty Analysis

**Figure 24:**
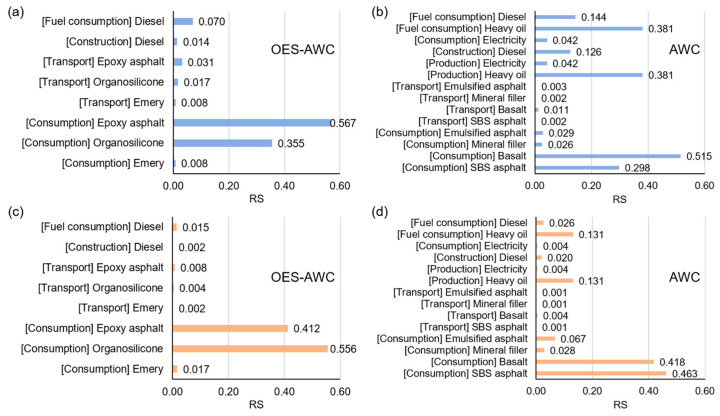
Relative sensitivity of (**a**) carbon emission of OES-AWC, (**b**) carbon emission of AWC, (**c**) cost of OES-AWC, and (**d**) cost of AWC.

Figure 24 delineates the relative sensitivity (RS) of various factors to the carbon emissions (CE) and lifecycle costs of both the OES-AWC and the conventional AWC. The analysis confirms that the consumption of primary raw materials—specifically asphalt binder and aggregates—exerts the most dominant influence on both environmental and economic outputs. Notably, owing to its composite modification strategy, the sensitivity profile of the OES-AWC diverges significantly from that of the conventional AWC. Within the OES-AWC system, the carbon emission sensitivity to epoxy asphalt reaches a substantial 0.567, while that to the organosilicon modifier stands at 0.355. These elevated values underscore the defining characteristic of high-performance functional additives: their low dosage yet exceptionally high specific carbon footprint per unit mass. This contrasts sharply with the conventional AWC, where sensitivities to basalt aggregate and SBS asphalt are distributed more uniformly at 0.418 and 0.463, respectively, indicating a reliance on bulk material volume. Consequently, while the conventional AWC is governed by the absolute quantity of its constituent materials, the OES-AWC is critically sensitive to the specific type and dosage of high-value modifiers. Based on these quantitative insights, the unit price of asphalt binder and the consumption of aggregates—the two predominant variables—have been identified as the core parameters for the subsequent Monte Carlo simulation, enabling a robust uncertainty analysis of the OES-AWC system under market fluctuations.

**Figure 25:**
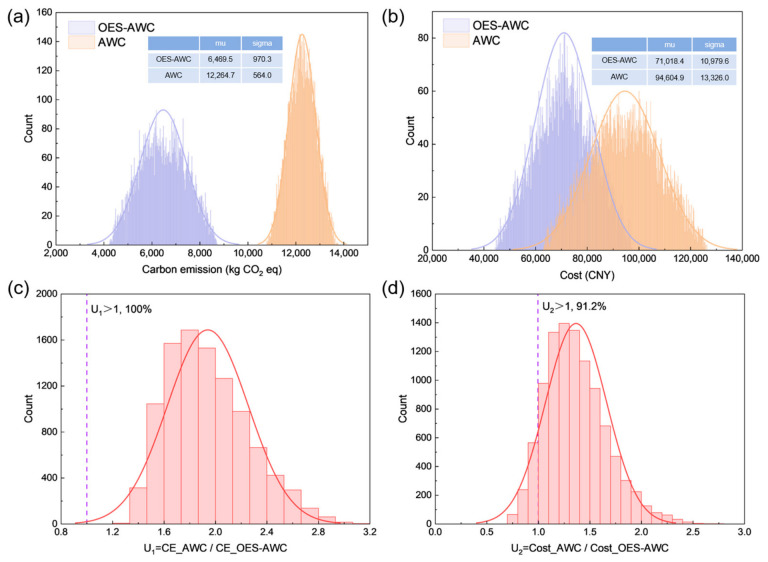
The probability distribution of carbon emission (**a**,**c**) and cost (**b**,**d**) of OES-AWC and AWC.

Figure 25 quantifies the uncertainty of environmental and economic benefits. The results indicate that the OES-AWC yields significantly lower mean carbon emissions and costs compared to the conventional AWC, with means of 6469.5 kg CO_2_ eq and 71,018.4 CNY, respectively, representing reductions of approximately 47% and 25% relative to the AWC. Crucially, the U_1_ ratio exceeds 1 with a probability of 100%, while the U_2_ ratio surpasses 1 with a probability of 91.2%. These statistical findings confirm that the carbon reduction and cost savings achieved by the OES-AWC are highly robust against parameter variations.

## 6. Conclusions

This study proposes a sustainable organosilicon-based erosion- and skid-resistant asphalt wearing course (OES-AWC) with enhanced durability and reduced life-cycle impacts, establishing a robust technical pathway for pavement maintenance in aggressive environments. The primary findings are summarized as follows:

(1) The organosilicon treatment forms a stable hydrophobic cross-linked network on the asphalt surface, leading to a substantial enhancement in resistance to multi-source environmental damage. The treated asphalt mixtures exhibit improved performance against hydrodynamic scouring, oil erosion, and freeze–shear action, with increases of 22.0%, 32.8%, and 41.1%, respectively, demonstrating its effectiveness in improving interfacial durability under complex service conditions.

(2) The optimized design of the wearing course, incorporating high-hardness emery aggregates, an appropriate binder–aggregate ratio, and a controlled organosilicon dosage, results in a surface structure with enhanced macro- and micro-texture characteristics. The system achieves a high initial skid resistance with a BPN of 82 and a texture depth of 1.06 mm, while maintaining structural integrity of the surface layer.

(3) Incorporating epoxy resin further enhances the structural stability of the wearing course by strengthening the bonding between aggregates and the asphalt matrix. This modification significantly improves resistance to mechanical wear and aggregate stripping, resulting in a 148.1% increase in wear durability and ensuring sustained functional performance under repeated loading.

(4) Within the defined “cradle-to-gate” system boundary, the OES-AWC demonstrates significant environmental and economic potential, achieving a 47.2% reduction in carbon emissions and a 25.0% decrease in total cost compared to conventional AWC. Probabilistic uncertainty analysis further confirms that these advantages are robust against fluctuations in raw material prices and consumption rates.

## 7. Limitations

While the OES-AWC demonstrated significant performance enhancements in resisting multi-source erosion and improving skid resistance within the laboratory framework, translating these findings into full-scale field applications necessitates stringent control over construction protocols and environmental parameters, specifically temperature and humidity.

## Figures and Tables

**Table 1:** Technical index of SBS-modified asphalt.

Technical Index	Unit	Tested Values
Penetration (25 °C)	0.1 mm	65
Ductility (5 °C, 5 cm/min)	mm	>100
Softening point (R&B)	°C	67.7
Viscosity (135 °C)	Pa·s	1.566

**Table 2:** Technical index of anti-skid aggregate.

Technical Index	Anti-Skid Aggregate	
	Basalt	Emery
Apparent density (g/cm^3^)	2.928	3.364
Water content (%)	0.02	0.02
Soundness (%)	4	3

**Table 3:** Technical index of waterborne epoxy resin and curing agent.

Technical Index	Epoxy Resin	Curing Agent
Epoxide value (mol/100 g)	0.53	N/A
Amine value (mg KOH/g)	N/A	1.46
Appearance	Transparent fluid	Dark yellow fluid
Tensile strength of cured epoxy resin (MPa)	1.63	
Fracture elongation of cured epoxy resin (%)	208	

**Table 4:** Performance test of skid-resistant fog seal and OES-AWC.

Performance	Test	Sample Size	Test Condition
Wear resistance	Wheel wearing	Φ 300 mm	25 °C
Skid resistance	British pendulum test	300 × 300 × 50 mm	25 °C
	Texture depth	300 × 300 × 50 mm	25 °C
Durability	Wheel tracking	300 × 300 × 50 mm	60 °C 0.7 MPa 42 times/min

**Table 5:** Fitting equation of spalling rate.

Organosilicon Content (g/m^2^)	Fitting Function	Equation Coefficient		R^2^
		*a*	*b*	
400	y=a·x+b	0.0034	31.7	0.9706
550	y=a·ln(x)+b	5.0206	−2.9412	0.9264
700		5.5832	−6.3579	0.9607
850		5.1504	0.8318	0.9507
1000		5.2803	4.6049	0.9358

**Table 6:** Fitting equation of skid resistance.

Skid-Resistance Index	Wearing Course	Fitting Function	Equation Coefficient		R^2^
			*a*	*b*	
BPN	AWC	y=a·x+b	−0.1407	71.345	0.9416
	OES-AWC		−0.0869	81.448	0.9882
TD (mm)	AWC		−0.0025	0.9176	0.9575
	OES-AWC		−0.0016	1.1338	0.9632

**Table 7:** Unit carbon emissions and costs of various energy sources and raw materials.

Energy and Materials Type	Equivalent CO_2_ Emission	Cost	Sources
Diesel	3.70 kg eq. CO_2_/kg	9820 CNY/t	Field investigation and *China Energy Statistical Year Book (2020)* [34]
Heavy oil	3.58 kg eq. CO_2_/kg	4300 CNY/t	
Electricity	0.97 kg eq. CO_2_/kWh	0.691 CNY/kWh	Field investigation and *Contribution of Working group II (IPCC)* [35]
SBS asphalt	393 kg eq. CO_2_/t	5000 CNY/t	Field investigation and Pianosi et al. [36]
Basalt	9.15 kg eq. CO_2_/t	170 CNY/t	Field investigation and J. Zou et al. [37]
Mineral filler	7.36 kg eq. CO_2_/t	240 CNY/t	Field investigation and Liu et al. [38]
Emulsified asphalt	233 kg eq. CO_2_/t	4200 CNY/t	Field investigation and *The Eurobitume Life-Cycle Inventory for Bitumen Version 3.1* [39]
Emery	13.4 kg eq. CO_2_/t	320 CNY/t	Field investigation
Organosilicon	874.6 kg eq. CO_2_/t	15,000 CNY/t	Field investigation
Epoxy asphalt	1630 kg eq. CO_2_/t	13,000 CNY/t	Field investigation and Song et al. [13]

**Table 8:** Unit asphalt pavement material input and material transportation distance.

Material Type	Material Input (ton)		Transpot Distance (km)
	OES-AWC	AWC	
SBS asphalt	0	8.64	30
Basalt	0	164.21	20
Mineral filler	0	8.64	35
Emulsified asphalt	0	1.5	40
Emery	3.79	0	65
Organosilicon	2.63	0	140
Epoxy asphalt	2.25	0	260

**Table 9:** Production and construction equipment of asphalt pavement.

Equipment	Energy Type	Energy Consumption Efficiency
Mixing equipment (Asphalt mixture production)	Heavy oil Electricity	6.95 kg/t 2.957 kWh/t
Dump truck (20 t) (Materials transport)	Diesel	0.21 kg/km
Mixture paver (Mixture paving)	Diesel	118.3 kg/km
Binder and aggregate distributor (Spraying organosilicon, aggregate, and epoxy resin)	Diesel	0.0023 kg/m^2^
Vibratory compactor (Pavement compaction)	Diesel	143.9 kg/km
Tire roller (Pavement compaction)	Diesel	121.3 kg/km

## Data Availability

The original contributions presented in this study are included in the article. Further inquiries can be directed to the corresponding authors.
